# Genome-wide blood DNA methylation analysis in patients with delayed cerebral ischemia after subarachnoid hemorrhage

**DOI:** 10.1038/s41598-020-68325-3

**Published:** 2020-07-10

**Authors:** Bong Jun Kim, Youngmi Kim, Dong Hyuk Youn, Jeong Jin Park, Jong Kook Rhim, Heung Cheol Kim, Keunsoo Kang, Jin Pyeong Jeon

**Affiliations:** 1 Institute of New Frontier Stroke Research, Hallym University College of Medicine, Chuncheon, Korea; 2 Department of Neurology, Konkuk University Medical Center, Seoul, Korea; 3 Department of Neurosurgery, Jeju National University College of Medicine, Jeju, Korea; 4 Department of Radioilogy, Hallym University College of Medicine, Chuncheon, Korea; 5 Department of Microbiology, College of Science & Technology, Dankook University, Cheonan, 31116 Korea; 6 Department of Neurosurgery, Hallym University College of Medicine, 77 Sakju-ro, Chuncheon, 24253 Korea; 7 Genetic and Research Inc., Chuncheon, Korea

**Keywords:** Medical research, Neurology

## Abstract

Little is known about the epigenetic changes associated with delayed cerebral ischemia (DCI) pathogenesis after subarachnoid hemorrhage (SAH). Here, we investigated genome-wide DNA methylation profiles specifically associated with DCI, which is a major contributor to poor clinical outcomes. An epigenome-wide association study (EWAS) and quantitative real-time PCR (qRT-PCR) were conducted in 40 SAH patients (DCI, n = 13; non-DCI, n = 27). A replication study using bisulfite modification and methylation-specific PCR was further performed in 36 patients (DCI, n = 12; non-DCI, n = 24). The relative degree of methylation was described as the median and 25th–75th percentile. No significant differences in clinical characteristics between DCI and non-DCI groups were observed. Among the top 10 differentially methylated genes analyzed via EWAS, two aberrantly methylated CpG sites of cg00441765 (*INSR* gene) and cg11464053 (*CDHR5* gene) were associated with decreased mRNA expression (2^−ΔCt^). They include *INSR* [0.00020 (0.00012–0.00030) in DCI vs. 0.00050 (0.00030–0.00068) in non-DCI] and *CDHR5* [0.114 (0.053–0.143) in DCI vs. 0.170 (0.110–0.212) in non-DCI]. Compared with non-DCI cases, patients with DCI exhibited an increased degree of methylation in the replication study: *INSR*, 0.855 (0.779–0.913) in DCI vs. 0.582 (0.565–0.689) in non-DCI; *CDHR5*, 0.786 (0.708–0.904) in DCI vs. 0.632 (0.610–0.679) in non-DCI. Hypermethylation of two novel genes, *INSR* and *CDHR5* may serve as a biomarker for early detection of DCI following SAH.

## Introduction

Delayed cerebral ischemia (DCI) is a major contributor to poor neurologic outcomes in patients diagnosed with subarachnoid hemorrhage (SAH)^1^. However, it is also a preventable and treatable complication. Well-known risk factors for DCI include female gender, cigarette smoking, hyperglycemia, high Hunt-Hess (HH) grade and thick hemorrhage at admission^1–3^. The majority of genetic studies investigating DCI have focused on gene expression or linkage analyses of candidate genes^4^. Candidate genes include those associated with inflammation, endothelial dysfunction, fibrinolysis, and brain metabolism. A previous meta-analysis^5^ showed that ApoE ε4 carriers had a higher risk of DCI than non- ε4 carriers. Kim et al.^6^ reported that DCI was more frequently observed in patients expressing haptoglobin (Hp) 2–2 than Hp 1–1 phenotype. In particular, SAH patients with Hp 2–1, and non-DCI patients showed higher α1intensities than DCI patients. A genome-wide association study (GWAS)^7^ revealed that SNP rs999662 encompassing solute carrier family 12 member 3 (*SLC12A3*) was significantly associated with high transcranial Doppler (TCD) velocities, such as angiographic vasospasm, which leads to DCI^7^.

Genetic background accounts for approximately 37.9% of stroke pathogenesis^8–10^. However, the frequency of susceptible genetic variants in stroke patients varies between 5 and 10%^9^, which indicates cryptic genetic changes beyond DNA sequences^8,11^. One of these cryptic variations involving novel epigenetic pathways is DNA methylation, histone modifications or non-coding RNA^11^. A positive correlation between higher methylation status of the inositol 1-,4-,5-trisphosphate receptor (*ITPR3*) and DCI development was observed in patients with SAH^11^. In their study, DCI patients manifested a lower expression of *ITPR3* mRNA concomitant with increased expression of DNA methyltransferase1 and a decrease in ten-eleven translocation methylcytosine dioxygenase 1 (*TET1*). Nevertheless, little is known about the epigenetic changes associated with DCI pathogenesis following SAH. Herein, we report an epigenome-wide association study (EWAS) comparing DNA methylation and DCI development to determine its role as a biomarker in DCI pathogenesis following SAH.

## Results

### Clinical characteristics of enrolled patients

Patient’s baseline characteristics are presented in Table 1. Variables such as female gender, HTN, DM, hyperlipidemia and smoking did not differ significantly between the DCI and non-DCI groups throughout the study period, although SAH patients in the discovery phase tended to be younger than those in the replication phase. In the discovery phase, higher HH grade was more frequently observed in DCI compared with non-DCI, but not statistical significant (p = 0.311). Most patients (80.0%) underwent coil embolization and 2 of 13 DCI patients (15.4%) underwent chemical angioplasty entailing intraarterial infusion of vasodilators during cerebral angiography to reverse cerebral vasospasm. In the replication phase, DCI patients appear to exhibit higher HH grade and Fisher grade, but was not statistically significant (p = 0.095 and p = 0.421, respectively). Most aneurysms were located in the anterior cerebral circulation.

**Table 1:** Baseline characteristics of subjects in discovery and replication phases.

Variables	Non-DCI (n = 27)	Discovery		Non-DCI (n = 24)	Replication	
		DCI (n = 13)	p-value		DCI (n = 12)	p-value
**Clinical findings**						
Female	15 (55.6%)	10 (76.9%)	0.398	17 (70.8%)	8 (66.7%)	0.801
Age, years	53.4 ± 12.2	53.2 ± 6.7	0.970	59.9 ± 8.3	58.4 ± 8.4	0.601
Hypertension	9 (33.3%)	2 (15.4%)	0.175	11 (45.8%)	7 (58.3%)	0.486
Diabetes mellitus	3 (11.1%)	0 (0.00%)	0.192	2 (8.3%)	2 (16.7%)	0.460
Hyperlipidemia	4 (14.8%)	1 (7.7%)	0.458	1 (4.2%)	1 (8.3%)	0.612
Smoking	5 (18.5%)	4 (30.8%)	0.505	2 (8.3%)	1 (8.3%)	1.000
**Radiologic findings**						
HH grade 4 and 5	7 (25.9%)	6 (46.2%)	0.311	7 (29.2%)	7 (58.3%)	0.095
Fisher grade 3 and 4	20 (74.1%)	12 (92.3%)	0.513	17 (70.8%)	10 (83.3%)	0.421
Anterior location	21 (77.8%)	11 (84.6%)	0.870	19 (79.2%)	9 (75.0%)	0.780
**Treatment**						
Coil embolization	22 (81.5%)	10 (76.9%)	0.326	19 (79.2%)	9 (75.0%)	0.780

*DCI* delayed cerebral ischemia, *HH* Hunt-Hess grade.

^a^Data are shown as the numbers of subjects (percentage) for discrete and categorical variables and mean ± standard deviation.

### Altered DNA methylation and mRNA expression in the discovery phase

**Figure 1:**
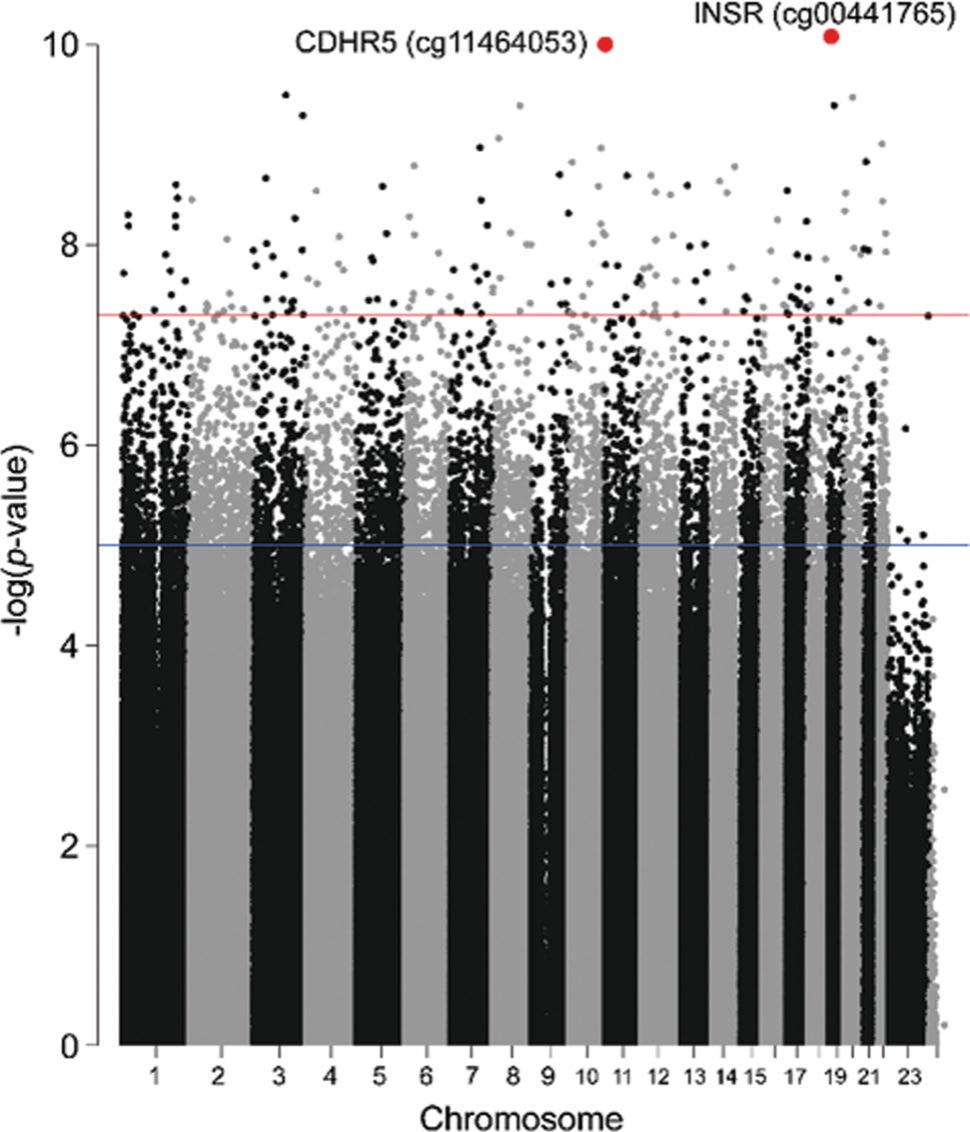
Manhattan plot of genome-wide DNA methylation analysis.

**Figure 2:**
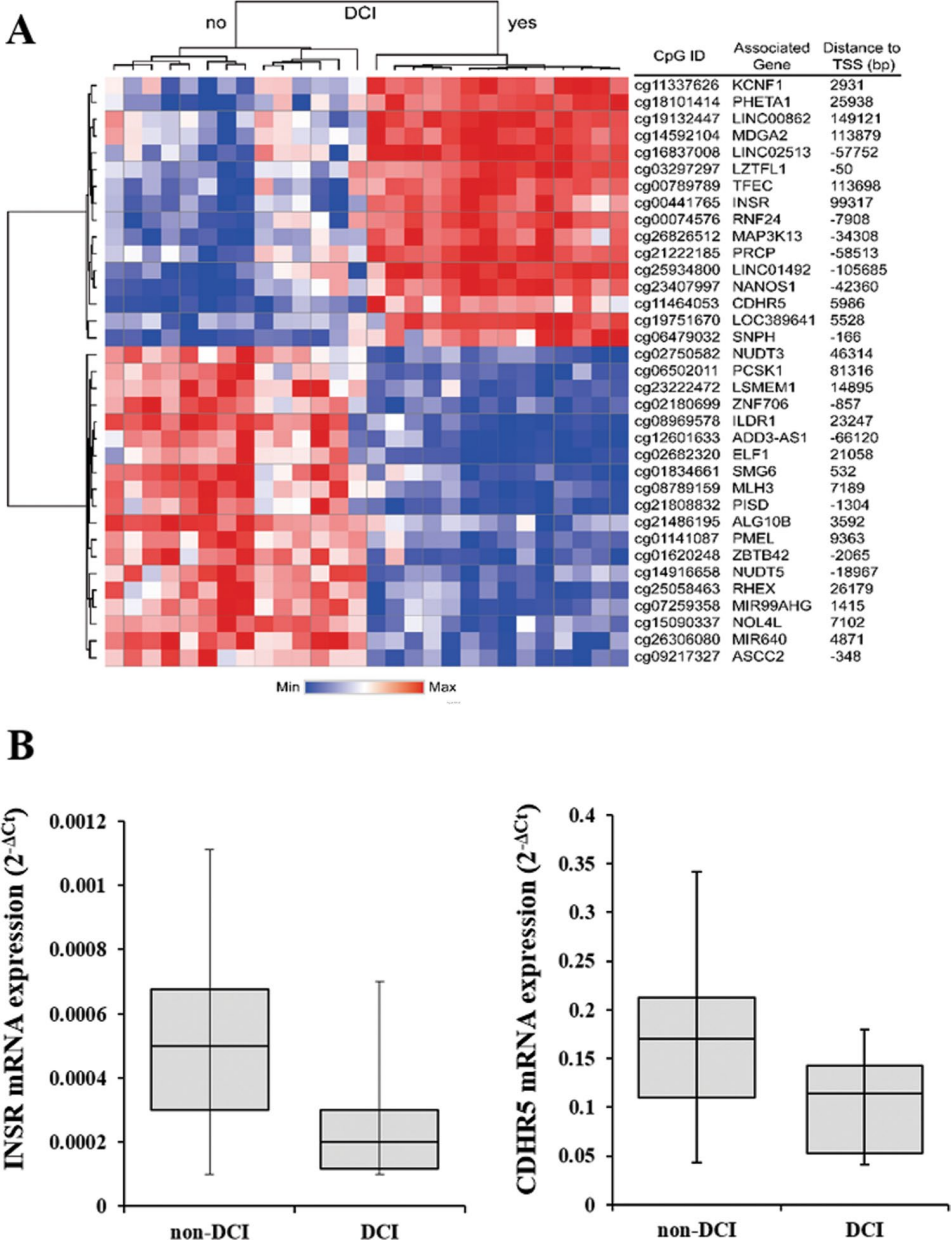
(**A**) Hierarchical clustering of differences in DNA methylation between delayed cerebral ischemia (DCI) and non-DCI patients. (**B**) Transcription analysis of the two differentially methylated gene candidates using qRT-PCR. The results showed a lower expression of *INSR* and *CDHR5* in DCI patients compared with non-DCI patients.

We evaluated significant epigenome-wide associations with DCI in 40 SAH patients. A total of 35 CpG sites passed the cutoff (Fig. 1). Among the differentially methylated CpG sites (DMCpGs) identified, cg00441765 and cg11464053 were the top two CpG sites showing hypermethylation in DCI patients compared with non-DCI patients (Table 2). Hierarchical clustering of CpG sites based on differences in DNA methylation in patients with DCI is presented in Fig. 2A. Interestingly, cg00441765 and cg11464053 were located in the second intron of insulin receptor (*INSR*) gene and in the exon 13 of the cadherin-related family member 5 (*CDHR5*).

**Table 2:** Methylation status and frequency of top 10 differentially methylated genes in delayed cerebral ischemia (DCI) and non-DCI patients following subarachnoid hemorrhage.

CpG site ID	Methylation in the DCI (vs non-DCI)	p-value	Q-value	Difference in beta value	Chromosome	Position	Distance to transcription start site (bp)	Gene name	Gene description
cg00441765	Hyper	8.55E−11	1.90E−05	0.0485	chr19	7,194,996	99,317	INSR	Insulin receptor
cg11464053	Hyper	1.01E−10	1.90E−05	0.0440	chr11	619,080	5,986	CDHR5	Cadherin related family member 5
cg08969578	Hypo	3.16E−10	2.54E−05	− 0.1450	chr3	121,717,880	23,247	ILDR1	Immunoglobulin like domain containing receptor 1
cg15090337	Hypo	3.32E−10	2.54E−05	− 0.0451	chr20	31,165,773	7,102	NOL4L	Nucleolar protein 4 like
cg02180699	Hypo	4.02E−10	2.54E−05	− 0.0858	chr8	102,219,149	− 857	ZNF706	Zinc finger protein 706
cg26306080	Hypo	4.01E−10	2.54E−05	− 0.0337	chr19	19,550,743	4,871	MIR640	MicroRNA 640
cg26826512	Hyper	5.05E−10	2.73E−05	0.0512	chr3	185,046,528	− 34,308	MAP3K13	Mitogen-activated protein kinase kinase kinase 13
cg09217327	Hypo	9.73E−10	3.68E−05	− 0.0126	chr22	30,234,641	− 348	ASCC2	Activating signal cointegrator 1 complex subunit 2
cg23222472	Hypo	1.06E−09	3.68E−05	− 0.1144	chr7	112,135,961	14,895	LSMEM1	Leucine rich single-pass membrane protein 1
cg19751670	Hyper	8.56E−10	3.68E−05	0.2183	chr8	23,088,262	5,528	LOC389641	Uncharacterized LOC389641

We evaluated the mRNA expression of the top 10 genes in 40 SAH patients via quantitative real-time PCR (qRT-PCR) analysis (Table 2). After exlcusion of the two genes, MIR640 (microRNA) and LOC389641 (long non-coding RNA), we further evaluated the relative mRNA expression (2^−ΔCt^) of 8 genes. DCI patients showed decreased transcription of *INSR* than in non-DCI patients (0.00020 [0.00012–0.00030] vs. 0.00050 [0.00030–0.00068]; p = 0.006). In addition, DCI patients showed lower *CDHR5* expression than the non-DCI patients (0.114 [0.053–0.143] vs. 0.170 [0.110–0.212]; p = 0.010) (Figs. 2B). Associations between the degree of methylation of the remaining six genes and their corresponding mRNA expression were not correlated significantly with other genes according to DCI development (Supplemental Table S2).

### MSP of *INSR* and *CDHR5* and mRNA expression in the replication phase

**Figure 3:**
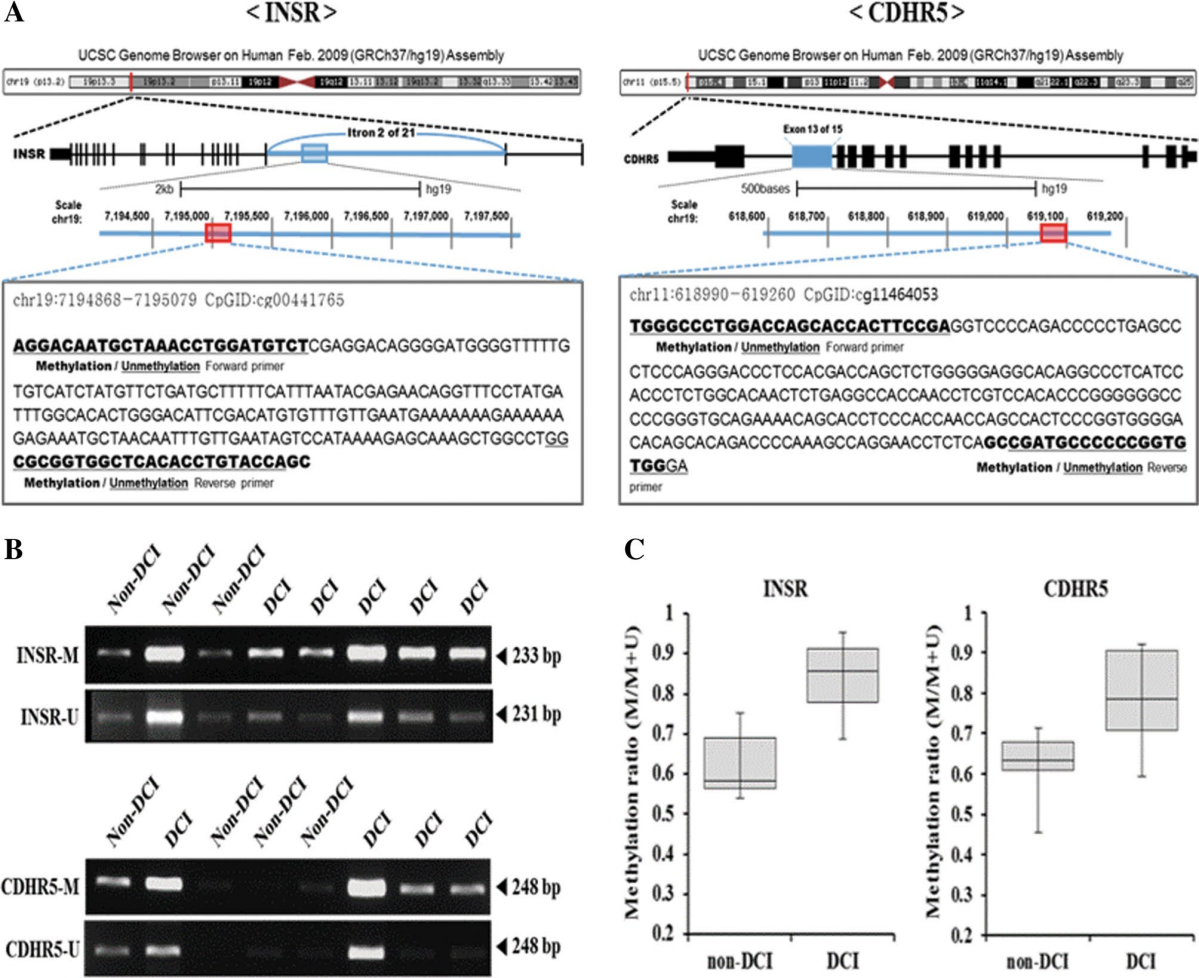
(**A**) Schematic representation of methylation-specific PCR (MSP) analysis associated with intron 13/15 above *INSR*, or exon 2/21 of the *CDHR5* genes. (**B**) Representative subsets of MSP of the *INSR* and *CDHR5* regions using methylated or unmethylated specific primer sets in 36 subarachnoid hemorrhage patients in the replication phase. (**C**) DCI patients show higher methylation level than non-DCI patients. Nucleotide sequences of *INSR* and *CDHR5*, and MSP primers are listed in supplemental data.

To evaluate the methylation status of selected *INSR* and *CDHR5* in DCI and non-DCI groups, the MSP primer sets were designed against the identical region annotated in Infinium MethylationEPIC assay (Fig. 3A) (Supplemental Table S1). The methylation level of two genes was measured using MSP in 36 patients with SAH in the replication phase (Figs. 3B,C)^11^. DCI patients exhibited a higher degree of methylation than non-DCI patients: *INSR*, 0.855 (0.779–0.913) in DCI vs. 0.582 (0.565–0.689) in non-DCI; p = 0.002; *CDHR5*, 0.786 (0.708–0.904) in DCI vs. 0.632 (0.610–0.679) in non-DCI; p = 0.017, respectively (Figs. 3A,B). Additionally, qRT-PCR was conducted to analyze the mRNA expression of the corresponding genes in 36 SAH patients. Patients with DCI had a lower level of *INSR* mRNA compared with those without DCI [0.00021 (0.00017–0.00024) vs. 0.00044 (0.00033–0.00065); p < 0.001]. DCI patients also expressed a lower level of *CDHR5* than non-DCI patients [0.121 (0.090–0.134) vs. 0.185 (0.151–0.229); p < 0.001] (Supplemental Fig. S3).

## Discussion

In this study, we identified two novel hypermethylated CpG sites of *INSR* and *CDHR5* in DCI patients following SAH. Our findings suggested that initial brain injury due to sudden surge in intracranial pressure (ICP) might affect DNA methylation patterns concomitant with decreased mRNA expression, resulting in DCI.

The *INSR* gene is distributed in the brain extensively, especially in choroid plexus, olfactory bulb and areas of the striatum and cerebral cortex. *INSR* signaling is associated with brain metabolism, maintaining neuronal function, synaptogenesis and mitochondrial activity^12^. Hancock et al.^13^ reported nuclear translocation of the insulin receptor from the cell surface leading to the formation of RNA polymerase II complex on chromatin. In particular, most *INSRs* bound to the promoters, suggesting regulation of genes linked to insulin function. Approximately, 75% of the SAH patients exhibited hyperglycemia at admission due to activated hypothalamic–pituitary–adrenal axis and increased inflammatory response^14,15^. Kruyt et al.^15^ reported that hyperglycemia is associated with DCI and poor clinical outcome in SAH patients. Hyperglycemia under ischemia–reperfusion injury can increase reactive oxygen species formation and anaerobic glycolysis associated with aggravation of infarct volume, disruption of homeostasis and subsequent brain injury^15^. However, the correlation between intensive insulin treatment and neurologic outcomes has yet to be firmly established^15^. To verify the correlation between DNA methylation of *INSR* (cg00441765) in the whole blood and brain, we used the UNAGE-CpG tool^16^. Spearman rho value of variable CpGs in the brain and blood was 0.64 (p = 0.0021, Supplemental Fig. S1), suggesting correlation of differentially expressed genes between the two tissues. Nevertheless, SAH severities at admission and intensive glucose monitoring via microdialysis to prevent hypoglycemia can skew the results. Therefore, studies investigating the effect of *INSR* dysfunction on DCI pathogenesis in vivo and in vitro are necessary, focusing on the advantages for patients with inhibited *INSR* methylation.

*CDHR5* is usually expressed in the kidney, liver and gastrointestinal tract. *CDHR5* is found mainly at the brush border of renal proximal tubules and intestinal epithelium^17,18^. *CDHR5* has been investigated as a biomarker for solid tumors such as colon cancer or renal cell carcinoma. Blasius et al.^17^ reported that *CDHR5* was expressed in approximately 75% of the renal cell carcinoma. Particularly, *CDHR5* expression was associated with a significantly longer survival time. In SAH, renal dysfunction has been reported in approximately 7% of the patients^19^. Zacharia et al.^20^ reported that SAH patients who were at risk for renal failure showed two-fold poor outcomes. Renal proximal tubule regulated blood pressure and plasma volume via sodium absorption in the glomerular filtrate^21^. Vrsajkov et al.^22^ reported that hyponatremia was associated with DCI and poor functional outcomes. In our study, DCI patients exhibited decreased transcription of *CDHR5* compared with non-DCI patients. In addition, hyponatremia was observed frequently in DCI patients. Nevertheless, various factors including SAH-induced sympathetic activation, contrast use, antibiotic therapy, surgical intervention, and aggressive management of fluid volume can increase the risk of renal failure^23^. Therefore, additional studies are needed to investigate the epigenetic effect of *CDHR5* on DCI, focusing on electrolyte imbalance.

In stroke research, epigenetic analysis has been performed in patients with ischemic stroke and atherosclerotic risk. Atherosclerotic aorta showed a higher degree of global DNA methylation of cytosine at CpG sites than in healthy human aorta^24^. Using EWAS, Rask-Andersen et al.^25^ reported myocardial infarction-specific methylation pattern in 211 CpG-sites of individuals living in northern Sweden. However, no significant stroke-associated methylation pattern was observed in 27 stroke patients^8^. Baccarelli et al.^26^ showed an association between hypomethylation and stroke occurrence or mortality. No significant difference in global methylation patterns was observed between stroke subtypes among patients with large-artery atherosclerosis, small-artery disease and cardio-aortic embolism^27^. Nevertheless, differences in biological age between stroke patients and healthy controls can affect the interpretation. Soriano-Tárraga et al.^28^ reported that ischemic stroke patients were biologically older than the healthy controls by an average age of 2.5 years. Due to variations in DNA methylation pattern in aging, a further epigenetic study investigating the role of age on stroke is required.

This study has some limitations. First, smoking status and sex were not included as covariates in the analysis due to the small sample size. Second, the expression of the two genes (*INSR* and *CDHR5*) was low, although the difference reached statistical significance. In addition, one non-DCI sample showed similar methylation pattern compared with the DCI sample. As a marker to distinguish DCI from non-DCI cases, the reliability of this CpG site should be further validated using in vivo and in vitro studies. Third, the difference in beta value is relatively less than expected. We have further analyzed the differences in beta value of all CpG sites via histogram (Supplemental Fig. S2). The results indicated that two CpG sites were significantly hypermethylated in DCI patients compared with non-DCI patients, although the difference in beta value was not dramatically altered. Fourth, we used bioinformatics tools to explore the molecular mechanisms of DNA methylation in DCI pathogenesis (Supplemental Table S3, Fig. S4). However, the proportion of the enrolled genes in the biological process and molecular pathway was small. Therefore, additional investigations involving a large cohort of SAH patients are further needed^11^.

## Conclusions

To the best of our knowledge, this is the first genome-wide epigenetic study analyzing the quantitative expression of the corresponding susceptibility genes to DCI following SAH. Two novel hypermethylation sites of *INSR* and *CDHR5* represent valuable biomarkers for the early detection of DCI. Studies investigating the precise mechanism underlying the methylation of candidate genes and DCI pathogenesis are further required.

## Methods and materials

### Study population

The derivation cohort was obtained from the stroke database of the Chuncheon Sacred Heart Hospital. The cohort is a prospective and observational project in the regional center of the district of Chuncheon city, the capital city of Gangwon Province in Korea^11,29–31^. In this database, we enrolled SAH patients with the following conditions: (1) adult SAH patients aged above 18 years; and (2) SAH due to ruptured aneurysm associated with saccular appearance. We excluded patients with the following conditions: (1) fusiform, dissection, traumatic and infectious aneurysms; (2) concomitant cerebrovascular diseases such as arteriovenous malformation or dural arteriovenous fistula; and (3) angiogram-negative SAH^32,33^. This study was performed in two phases: discovery and replication. In the discovery phase, the susceptible epigenetic marker was identified in 40 patients with SAH from September 2016 to October 2017 using EWAS. In the replication phase, an independent cohort of 36 patients between September 2017 and July 2019 were subjected to methylation-specific PCR.

We investigated the epigenetic patterns of blood representing potential markers for the prediction of DCI after SAH. DCI was defined by new neurologic deficits including motor weakness, sensory changes, dysphasia and decreased level of consciousness with concomitant cerebral vasospasm^6,11^. Clinical demographics regarding gender, age, hypertension (HTN), diabetes mellitus (DM), hyperlipidemia, smoking, and aneurysms detected radiologically were reviewed. This study was approved by the Institutional Review Board (No. 2016-3, 2017-9 and 2018-6) and informed consent was obtained from the patients or their relatives.

### Extraction and quantification of genomic DNA

Genomic DNA (gDNA) was extracted from buffy coat, which was collected by centrifuging whole blood samples at 3,000×*g* for 4 min at room temperature. The buffy coat fractions were stored at − 80 ℃ until ready for further processing. The gDNA was extracted from buffy coat with FlexiGene DNA kit (Qiagen, Hilden, Germany) according to the manufacturer’s instructions. The concentration and purity of gDNA were determined by measuring the absorbance ratio (A260/A280) using UV Eppendorf BioSpectrometer (Eppendorf, Hamburg, Germany)^11^.

### Epigenome-wide association study

In the discovery phase, we evaluated genome-scale DNA methylation profiles. Genomic DNA was analyzed using the Infinium MethylationEPIC (EPIC) assay with sodium bisulfite using the EZ-96 DNA methylation kit obtained from Zymo Research (CA, USA) according to manufacturer’s instructions. DNA methylation was quantified using the Infinium MethylationEPIC BeadChip (Illumina, CA, USA). Potentially existing raw quality probes in the raw data were filtered by the *minfi* package (version 1.24.0)^34^. First, samples with non-significant mean detection p-values (> 0.05) were excluded. The functional normalization algorithm (preprocessFunnorm) for Illumina methylation microarrays implemented in the *minifi* package was used to eliminate undesirable variation by regressing out variability with the control probes present in our methylation microarray. Finally, the differentially methylated CpG sites (DMCpGs) were identified with a q-value cutoff of 0.00005. To report methylation levels, the beta (β) value, which is an estimate of methylation level based on the ratio of intensity between methylated and unmethylated alleles, was calculated for each CpG site.

### Quantitative real-time PCR

Total RNA was extracted from fresh whole blood using QIAamp RNA Blood Mini Kit (Qiagen, Hilden, Germany) according to the manufacturer’s instructions. RNA quality and quantity were determined using the UV Eppendorf BioSpectrometer (Eppendorf, Hamburg, Germany). Isolated RNA (2 µg) was used for cDNA synthesis using Maxime RT PreMix Kit (iNtRON Biotechnology, Korea). The gene expression level was measured by quantitative real-time PCR (qRT-PCR) using the Power SYBR Green PCR master Mix (Thermo Fisher Scientific, MA, USA). PCR was performed for 45 cycles under the following conditions: 94 °C for 15 s, 55 °C for 30 s, and 70 °C for 30 s in the Rotor-Gene Q (Qiagen, Hilden, Germany). The qRT-PCR primers used in this study are listed in the supplemental data. Actin was used as an internal reference gene.

### Methylation-specific PCR

In the replication phase, methylation-specific PCR (MSP) was further performed after selecting the two candidate genes. Approximately, 2 µg of DNA was subjected to bisulfite modification using the EpiTect Bisulfite Kit (Qiagen, Hilden, Germany) according to the manufacturer’s instructions^11^. The modified DNA was subjected to MSP^11,35^. MSP primer sets were designed using the MethPrimer (https://www.urogene.org/methprimer/index1.html). The primer sequences, product sizes and target regions of PCR amplification are described in the supplemental data. Forty-five cycles of PCR using both unmethylated and methylated primers were performed under the following conditions: 95 °C for 15 s, 55 °C for 15 s and 72 °C for 30 s using 1.25 U Taq (iTaq, Intron Biotechnology, Seoul, Korea) in a final volume of 20 µl. PCR products were analyzed by electrophoresis on 3% agarose gel and visualized on UV.

### Statistical analysis

Differentially methylated CpG sites (DMCpGs) were identified with an adjusted p-value cutoff of 0.00005^36^. To compare the methylation profiles between the DCI and non-DCI groups, the scatter plots for probe methylation values and the box plots for genomic features of each group were drawn by in-house R scripts. The relative methylation intensities are expressed as medians with interquartile range^6^. Relative methylation intensity was calculated by dividing the degree of methylation by the combined intensity of methylation and unmethylation^11^. The degree of relative methylation and mRNA expression were compared using the Mann–Whitney U test. p-value < 0.05 was considered statistically significant. The analysis was performed using MedCalc software (Medcalc, Mariakerke, Belgium).

### Ethical approval

Sample collection and study design were performed according to the principles of the Declaration of Helsinki and were approved by Coordinating Ethnics Committee of the Chuncheon Sacred Heart Hospital.

## Supplementary information

Supplementary Information

## Data Availability

Data are available from the corresponding author (JPJ) upon ethical approval from the IRB of the participating hospital.

## References

[1] Duan W (2018). Risk factors and clinical impact of delayed cerebral ischemia after aneurysmal subarachnoid hemorrhage: Analysis from the china national stroke registry. Neuroepidemiology.

[2] Nahed BV (2007). Genetics of intracranial aneurysms. Neurosurgery.

[3] Geraghty JR, Testai FD (2017). Delayed cerebral ischemia after subarachnoid hemorrhage: Beyond vasospasm and towards a multifactorial pathophysiology. Curr. Atheroscler. Rep..

[4] Ducruet AF (2010). Genetic determinants of cerebral vasospasm, delayed cerebral ischemia, and outcome after aneurysmal subarachnoid hemorrhage. J. Cereb. Blood Flow Metab..

[5] Hu X (2018). Role of apolipoprotein e genotypes in aneurysmal subarachnoid hemorrhage: Susceptibility, complications, and prognosis. World Neurosurg..

[6] Kim BJ, Kim Y, Kim SE, Jeon JP (2018). Study of correlation between hp alpha1 expression of haptoglobin 2–1 and clinical course in aneurysmal subarachnoid hemorrhage. World Neurosurg..

[7] Kim H (2013). Cerebral vasospasm after sub-arachnoid hemorrhage as a clinical predictor and phenotype for genetic association study. Int. J. Stroke.

[8] Krupinski J (2018). DNA methylation in stroke. Update of latest advances. Comput. Struct. Biotechnol. J..

[9] Bevan S (2012). Genetic heritability of ischemic stroke and the contribution of previously reported candidate gene and genomewide associations. Stroke.

[10] Domingues-Montanari S, Mendioroz M, del Rio-Espinola A, Fernandez-Cadenas I, Montaner J (2008). Genetics of stroke: A review of recent advances. Expert Rev. Mol. Diagn..

[11] Kim BJ (2019). Correlation between altered DNA methylation of intergenic regions of itpr3 and development of delayed cerebral ischemia in subarachnoid hemorrhage patients. World Neurosurg..

[12] Kleinridders A, Ferris HA, Cai W, Kahn CR (2014). Insulin action in brain regulates systemic metabolism and brain function. Diabetes.

[13] Hancock ML (2019). Insulin receptor associates with promoters genome-wide and regulates gene expression. Cell.

[14] Kruyt ND, Biessels GJ, Devries JH, Roos YB (2010). Hyperglycemia in acute ischemic stroke: Pathophysiology and clinical management. Nat. Rev. Neurol..

[15] Kruyt ND (2010). Hyperglycemia in aneurysmal subarachnoid hemorrhage: A potentially modifiable risk factor for poor outcome. J. Cereb. Blood Flow Metab..

[16] Spindola LM (2019). Detecting multiple differentially methylated cpg sites and regions related to dimensional psychopathology in youths. Clin. Epigenet..

[17] Blasius FM (2017). Loss of cadherin related family member 5 (cdhr5) expression in clear cell renal cell carcinoma is a prognostic marker of disease progression. Oncotarget.

[18] Crawley SW (2014). Intestinal brush border assembly driven by protocadherin-based intermicrovillar adhesion. Cell.

[19] Solenski NJ (1995). Medical complications of aneurysmal subarachnoid hemorrhage: A report of the multicenter, cooperative aneurysm study. Participants of the multicenter cooperative aneurysm study. Crit. Care Med..

[20] Zacharia BE (2009). Renal dysfunction as an independent predictor of outcome after aneurysmal subarachnoid hemorrhage: A single-center cohort study. Stroke.

[21] Horita S (2017). The role of renal proximal tubule transport in the regulation of blood pressure. Kidney Res. Clin. Pract..

[22] Vrsajkov V, Javanovic G, Stanisavljevic S, Uvelin A, Vrsajkov JP (2012). Clinical and predictive significance of hyponatremia after aneurysmal subarachnoid hemorrhage. Balkan Med. J..

[23] Chen S (2014). The harmful effects of subarachnoid hemorrhage on extracerebral organs. Biomed. Res. Int..

[24] Zaina S (2014). DNA methylation map of human atherosclerosis. Circ. Cardiovasc. Genet..

[25] Rask-Andersen M (2016). Epigenome-wide association study reveals differential DNA methylation in individuals with a history of myocardial infarction. Hum. Mol. Genet..

[26] Baccarelli A (2010). Ischemic heart disease and stroke in relation to blood DNA methylation. Epidemiology..

[27] Soriano-Tarraga C (2014). Global DNA methylation of ischemic stroke subtypes. PLoS ONE.

[28] Soriano-Tarraga C (2016). Ischemic stroke patients are biologically older than their chronological age. Aging (Albany, N.Y.).

[29] Hong EP (2019). Genomic variations in susceptibility to intracranial aneurysm in the Korean population. J. Clin. Med..

[30] Kim CH, Jeon JP, Kim SE, Choi HJ, Cho YJ (2018). Endovascular treatment with intravenous thrombolysis versus endovascular treatment alone for acute anterior circulation stroke: A meta-analysis of observational studies. J. Korean Neurosurg. Soc..

[31] Cho YD, Kim SE, Lim JW, Choi HJ, Cho YJ, Jeon JP (2018). Protected versus unprotected carotid artery stenting: Meta-analysis of the current literature. J. Korean Neurosurg. Soc..

[32] Hong EP (2017). A novel association between lysyl oxidase gene polymorphism and intracranial aneurysm in Koreans. Yonsei Med. J..

[33] Hong EP, Kim BJ, Kim C, Choi HJ, Jeon JP (2018). Association of sox17 gene polymorphisms and intracranial aneurysm: A case-control study and meta-analysis. World Neurosurg..

[34] Aryee MJ (2014). Minfi: A flexible and comprehensive bioconductor package for the analysis of infinium DNA methylation microarrays. Bioinformatics.

[35] Herman JG, Graff JR, Myohanen S, Nelkin BD, Baylin SB (1996). Methylation-specific pcr: A novel pcr assay for methylation status of cpg islands. Proc. Natl. Acad. Sci. U.S.A..

[36] Pera J (2013). Gene expression profiling of blood in ruptured intracranial aneurysms: In search of biomarkers. J. Cereb. Blood Flow Metab..

