# Supplementary material for: Genome-wide blood DNA methylation analysis in patients with delayed cerebral ischemia after subarachnoid hemorrhage
Source: Sci Rep. 2020 Jul 10;10:11419. doi: 10.1038/s41598-020-68325-3 (PMC7351711; doi:10.1038/s41598-020-68325-3)

**Supporting Information**

**Genome-wide blood DNA methylation analysis in patients with delayed cerebral ischemia after subarachnoid hemorrhage**

Bong Jun Kim,^1*^ Youngmi Kim,^1*^ Dong Hyuk Youn,^1^ Jeong Jin Park,^2^ Jong Kook Rhim,^3^ Heung Cheol Kim,^4^ Keunsoo Kang,^5^ Jin Pyeong Jeon^1,6,7^

^1^Institute of New Frontier Stroke Research, Hallym University College of Medicine, Chuncheon, Korea

^2^Department of Neurology, Konkuk University Medical Center, Seoul, Korea

^3^Department of Neurosurgery, Jeju National University College of Medicine, Jeju, Korea

^4^Department of Radioilogy, Hallym University College of Medicine, Chuncheon, Korea

^5^Department of Microbiology, College of Science & Technology, Dankook University, Cheonan, Korea

^6^Department of Neurosurgery, Hallym University College of Medicine, Chuncheon, Korea

^7^Genetic and Research Inc., Chuncheon, Korea

**Table S1.** Primer sequences, product size and target position.

| **Name** | **Sequence ( 5' -> 3' )** | **Product**  **Size (bp)** | **Position^c^** |
| --- | --- | --- | --- |
| ***MSP primers*** | | | |
| INSR-MF^a^ | GGATAATGTTAAATTTGGATGTTTC | 233 | chr19:7194996  chr19:7194868-7195079 |
| INSR-MR | ACTAATACAAATATAAACCACCGCG |  |  |
| INSR-UF^b^ | GGATAATGTTAAATTTGGATGTTTT | 231 |  |
| INSR-UR | TAATACAAATATAAACCACCACACC |  |  |
| CDHR5-MF | TGGGTTTTGGATTAGTATTATTTTCGA | 248 | chr11:619080  chr11:618990-619260 |
| CDHR5-MR | CCACACCGAAAAACATCGAC |  |  |
| CDHR5-UF | TGGGTTTTGGATTAGTATTATTTTTGA | 248 |  |
| CDHR5-UR | TCCCACACCAAAAAACATCAAC |  |  |
| ***qRT-PCR primers*** | | | |
| INSR-F | AGACGTCCCGTCAAATATTGC | 341 | 3331 – 3351 |
| INSR-R | ACTCGTTGACCGTCTTCACC | 341 | 3671 - 3652 |
| CDHR5-F | GAGGGAGAGGTTGTGCTGAC | 281 | 1287 – 1306 |
| CDHR5-R | CTCAGAGTTGTGCCAGAGGG | 281 | 1567 - 1548 |

^a^ M indicates the primer for methylated alleles

^b^ U denotes the primer for unmethylated alleles

^C^ Primer positions of qRT-PCR represent the base from the start codon. The first nucleotide of the start codon is defined as position 1.

**Table S2.** The mRNA expression profiles of the six out of 10 aberrant methylated candidate genes in patients with and without delayed cerebral ischemia (DCI) following subarachnoid hemorrhage.

| Gene name | DCI (2^-ΔCt^) | non-DCI (2^-ΔCt^) | *p-value* |
| --- | --- | --- | --- |
| *ILDR1* | 0.0387 (0.0348–0.0523) | 0.0555 (0.0373–0.0746) | 0.076 |
| *NOL4L* | 0.0044 (0.0028–0.0063) | 0.0074 (0.0039–0.0095) | 0.071 |
| *ZNF706* | 0.0060 (0.0050–0.0077) | 0.0068 (0.0062–0.0083) | 0.170 |
| *MAP3K13* | 0.0802 (0.0618–0.0917) | 0.0909 (0.0728–0.101) | 0.130 |
| *ASCC2* | 0.0004 (0.0002–0.0010) | 0.0009 (0.0007–0.0011) | 0.170 |
| *LSMEM1* | 0.0167 (0.0104–0.0288) | 0.0260 (0.0145–0.0347) | 0.129 |

**Fig. S1.** Spearman correlation and p-value of methylation between the brain and the blood using the UNAGE-CpG tool (<http://han-lab.org/methylation/default/imageCpG>).


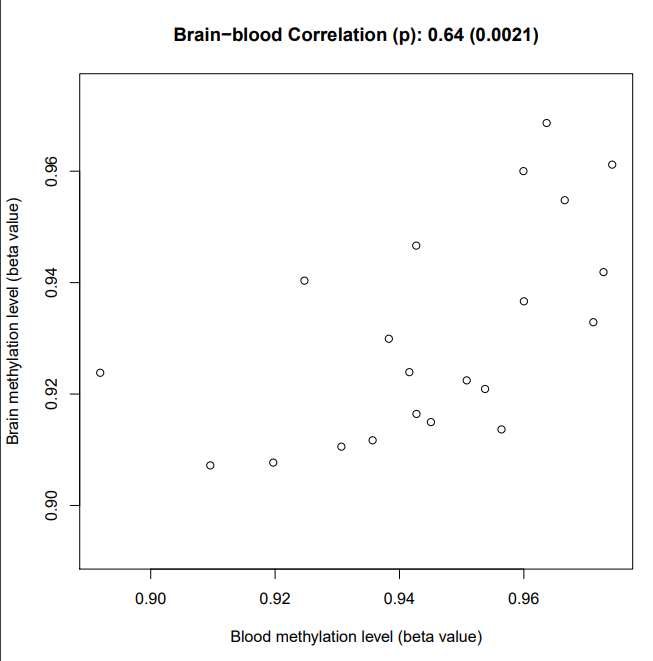


**Fig. S2.** The histogram showing difference in beta-value of the interrogated CpG sites in total (n = 865859) according to delayed cerebral ischemia following subarachnoid hemorrhage.

**
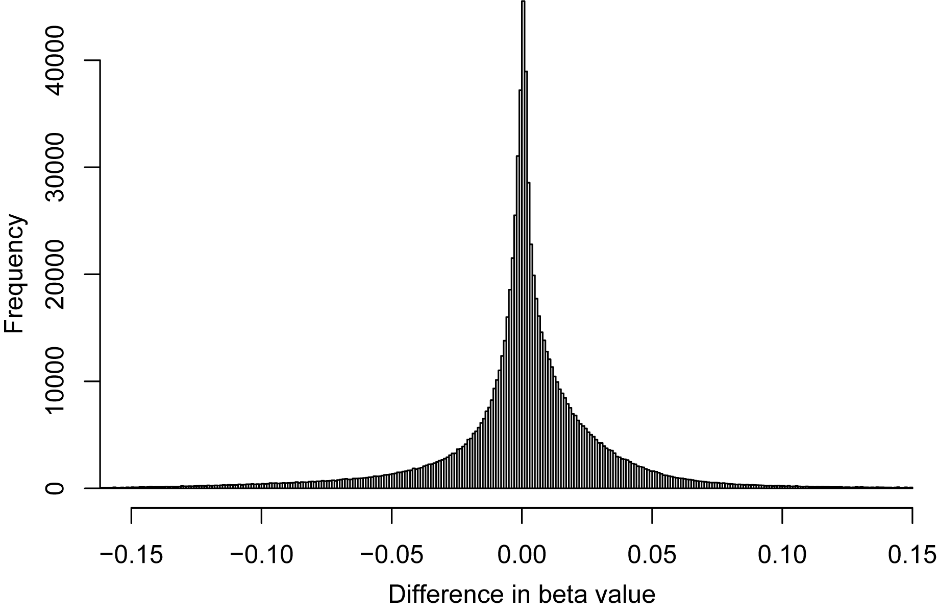
**

**Fig. S3.** Quantitative RT-PCR analysis of mRNA expression of *INSR* and *CDHR5*. Patients with DCI showed a lower expression of *INSR* and *CDHR5* than those without DCI in the replication phase.


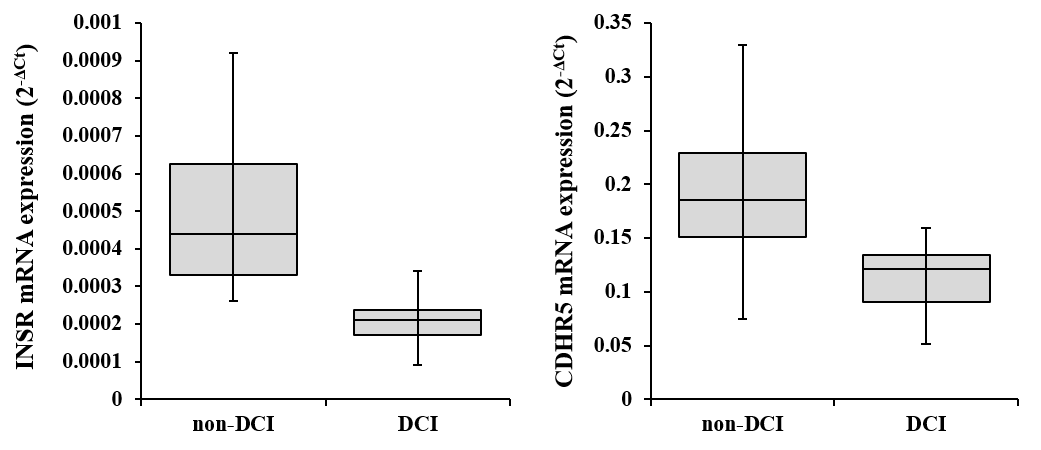


**Functional pathway enrichment and protein-protein interaction analysis**

Gene ontology (GO) and the Kyoto Encyclopedia of Genes and Genomes (KEGG) pathway enrichment analyses involved the top 200 differentially methylated genes in DCI and non-DCI patients using DAVID bioinformatics resources. P-value < 0.05 was regarded as statistically significant. Enriched biological processes (BP) included positive regulation of GTPase activity, thymus development, and microspike assembly. KEGG pathway analysis showed enrichment in 5 pathways including cancer, metabolic, endocytosis, MAPK signaling, and PI3K-Akt (Supplemental Table 3).

We further constructed the protein-protein interaction (PPI) networks using the STRING. The cut-off standard was based on an interaction score of 0.4 (Supplemental Figure 4). The four clusters and their hub genes involved in the PPI network were *TFDP1, RHOBTB1, HSPA8*, and *SREBF1*.

**Table S3.** GO and KEGG pathway enrichment analysis of differentially methylated genes.

| **Term** | **Count** | **%** | **P-value** | **Genes** |
| --- | --- | --- | --- | --- |
| **GO TERM_BP_DIRECT** |  |  |  |  |
| GO:0043547~positive regulation of GTPase activity | 11 | 0.047 | 0.012 | *DIS3, MCF2L2, LIMS1, DOCK1, RAP1GAP, RASGRP1, ARHGAP15, DENND3, DENND1B, FGD4, RIN3* |
| GO:0045892~negative regulation of transcription, DNA-templated | 8 | 0.034 | 0.09 | *ATXN1, ZNF706, LIMS1, NACC2, YAF2, JARID2, HSPA8, CBX5* |
| GO:0001934~positive regulation of protein phosphorylation | 4 | 0.017 | 0.074 | *PRR5, RASGRP1, ABL1, INSR* |
| GO:0030036~actin cytoskeleton organization | 4 | 0.017 | 0.079 | *PACSIN2, ABL1, CAPZB, FGD4* |
| GO:0051056~regulation of small GTPase mediated signal transduction | 4 | 0.017 | 0.084 | *RAP1GAP, RHOBTB1, ARHGAP15, FGD4* |
| GO:0048538~thymus development | 3 | 0.013 | 0.043 | *HOXA3, JARID2, ABL1* |
| GO:0043647~inositol phosphate metabolic process | 3 | 0.013 | 0.051 | *NUDT3, PLCH1, INPP4B* |
| GO:0030035~microspike assembly | 2 | 0.009 | 0.038 | *ABL1, FGD4* |
| GO:1902715~positive regulation of interferon-gamma secretion | 2 | 0.009 | 0.06 | *RASGRP1, ABL1* |
| GO:2000773~negative regulation of cellular senescence | 2 | 0.009 | 0.081 | *CDK6, ABL1* |
| GO:0034329~cell junction assembly | 2 | 0.009 | 0.088 | *LIMS1, GRHL2* |
| GO:0032801~receptor catabolic process | 2 | 0.009 | 0.088 | *SMURF1, KIF16B* |
| GO:0045995~regulation of embryonic development | 2 | 0.009 | 0.095 | *INSR, NOCT* |
| **KEGG pathway** |  |  |  |  |
| hsa01100:Metabolic pathways | 8 | 0.434 | 9.16E-04 | *ALG10B, UPB1, PLCH1, INPP4B, PISD, ATP6V0A4, GALNT8, LIPF* |
| hsa05200:Pathways in cancer | 5 | 0.563 | 9.66E-06 | *VHL, RASGRP1, CDK6, LAMC1, ABL1* |
| hsa04144:Endocytosis | 4 | 0.543 | 3.55E-05 | *RAB11FIP2, SMURF1, CAPZB, HSPA8* |
| hsa04010:MAPK signaling pathway | 3 | 0.42 | 0.003 | *RASGRP1, MAP3K13, HSPA8* |
| hsa04151:PI3K-Akt signaling pathway | 3 | 0.429 | 0.006 | *CDK6, LAMC1, INSR* |

**Fig. S4**. Protein-protein interaction network of differentially methylated genes in delayed cerebral ischemia (DCI) and non-DCI patients following subarachnoid hemorrhage
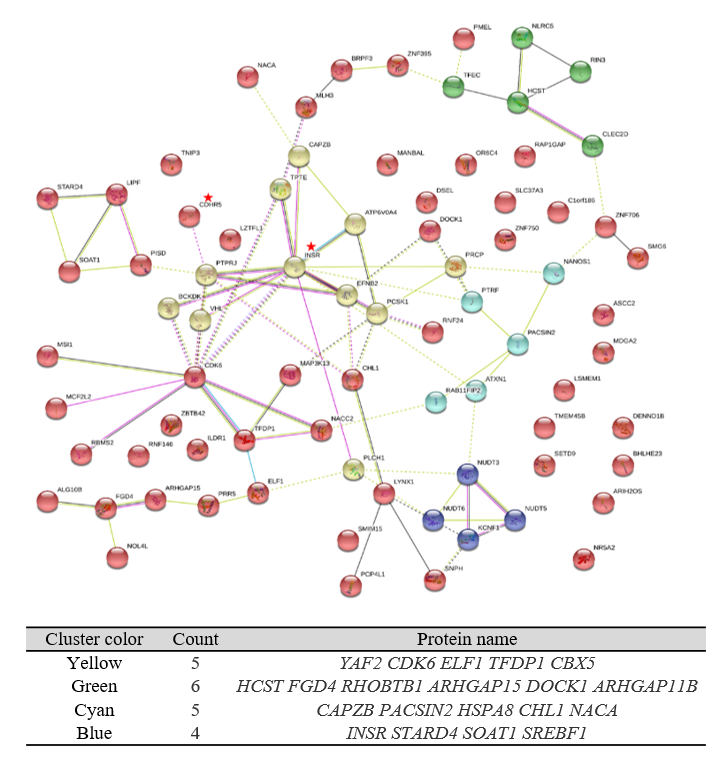


**Fig.** Original scans of the Figure 3B in the manuscript.


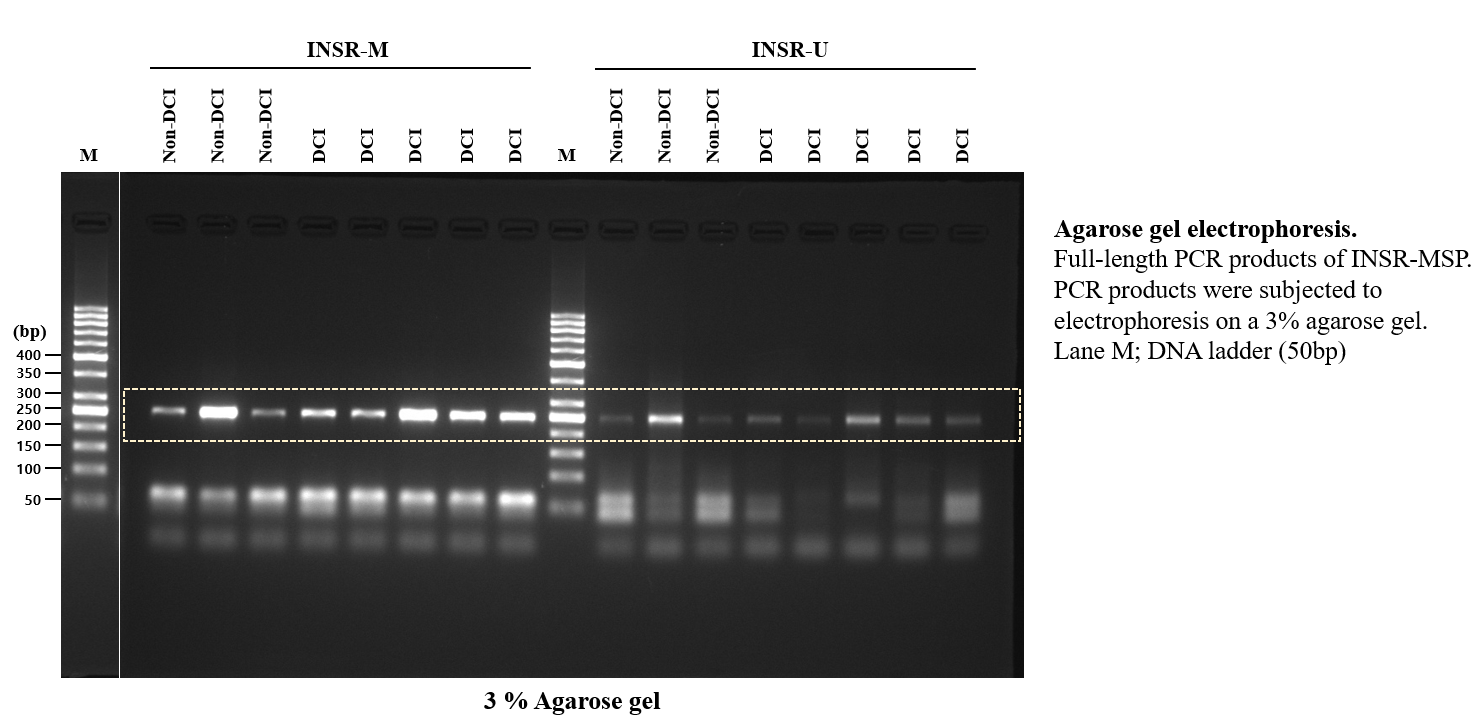


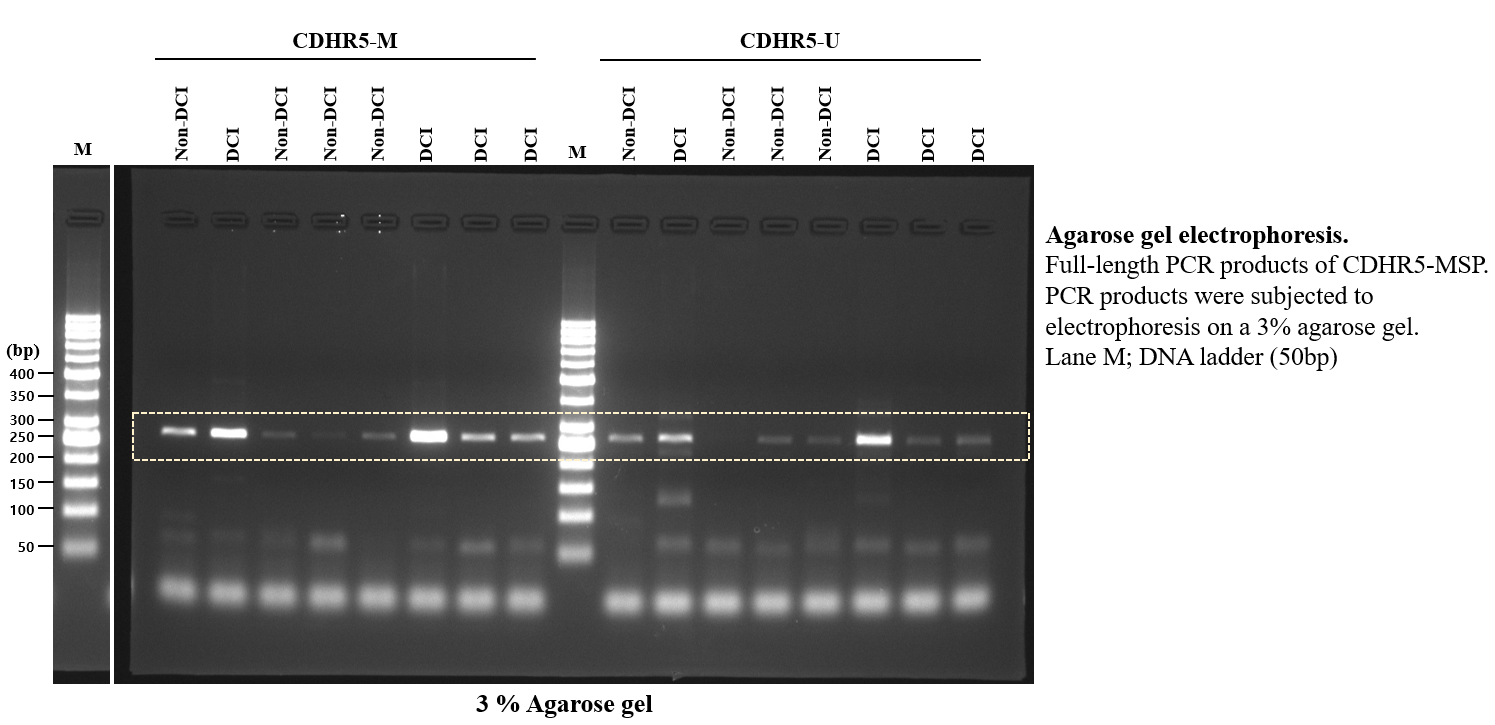

Supplement: Supplementary file 1 — Supplementary Information [file 41598_2020_68325_MOESM1_ESM.docx]
